# Process-oriented training in breastfeeding for health professionals decreases women’s experiences of breastfeeding challenges

**DOI:** 10.1186/1746-4358-9-15

**Published:** 2014-09-09

**Authors:** Ingrid Blixt, Lena B Mårtensson, Anette C Ekström

**Affiliations:** 1 Department of Obstetrics and Gynaecology, Eskilstuna, Mälarhospital, Sweden; 2 School of Health and Education, University of Skövde, Skövde, Sweden

**Keywords:** Process-oriented training, Support, Health professionals, Counselling, Breastfeeding-problems, Intervention study

## Abstract

**Background:**

The World Health Organization recommends promoting exclusive breastfeeding for six months. Women often end breastfeeding earlier than planned, however women who continue to breastfeed despite problems often experience good support and counselling from health professionals. The aim of this study was to evaluate the effects of a process-oriented training in breastfeeding support counselling for midwives and child health nurses, on women’s satisfaction with breastfeeding counselling, problems with insufficient breast milk and nipple pain in relation to exclusive breastfeeding shorter or longer than 3 months.

**Methods:**

An intervention through process-oriented training for health professionals regarding support in childbearing and breastfeeding took part in the south west of Sweden. This study was conducted in Sweden, in 2000 - 2003. Ten municipalities were paired, and within each pair, one was randomly assigned to the group of five intervention (IG) municipalities and one to the group of five control municipalities. Primiparas (n = 540) were invited to participate in a longitudinal study to evaluate the care they received. A survey was distributed at 3 days, 3 months and 9 months postpartum. Data collection for control group A (n = 162) started before the intervention was initiated. Data for control group B (n = 172) were collected simultaneously with the intervention group (IG) (n = 206). Women were also divided into two groups depending on whether they exclusive breastfed < 3 months or ≥ 3 months.

**Results:**

Women in IG were more satisfied with the breastfeeding counselling (p = 0.008) and felt the breastfeeding counselling was more coherent (p = 0.002) compared to control groups, when exclusive breastfeeding was < 3 months. In addition fewer women in the IG, among the group exclusively breastfeeding < 3 months, had problems with insufficient breast milk compared to the control groups (p = 0.01).

**Conclusion:**

A process-oriented training for health professionals in support influenced women’s ability to solve breastfeeding problems such as the experience of insufficient breast milk production. Women with exclusive breastfeeding lasting ≥ 3 months more often had breastfeeding duration in line with their planned breastfeeding duration, compared to women who had breastfeeding duration < 3 months.

**Trial registration:**

ACTRN12611000354987

## Background

Studies show health benefits of breastfeeding for children in developed countries, for both the women and child [1-3]. If children are breastfed exclusively, for at least three months, the cost of healthcare during their first year of life can be remarkably decreased [4]. The World Health Organization (WHO) recommends exclusive breastfeeding for the first six months of life. From six months of age, WHO suggests that solids should be introduced as a complement to breast milk, and recommends breastfeeding for two years or longer [5]. Women who want to breastfeed are often motivated to get through breastfeeding difficulties and breastfeed as long as they planned [6]. Women who ended their breastfeeding earlier than they planned often expressed disappointment, sadness and regret over not being able to breastfeed [6]. Further, the women often decide whether to breastfeed or not in late pregnancy. These women often have a negative attitude towards breastfeeding and had low confidence in their ability to breastfeed [7]. The self-confidence is often moderated by the experience of getting support [8]. Support from partner and grandmothers [8,9] as well as professional support [8,10] has a positive impact on women’s ability to breastfeed [8-10]. Lack of professional support has a negative impact on women’s ability to breastfeed [7,11].

Health professionals have difficulty providing good support when they lack time and evidence-based knowledge and when they have negative attitudes towards breastfeeding. These often result in contradictory breastfeeding advice [12]. Health professionals often give conflicting advice about breastfeeding on demand [13], length and timing of feedings [14], supplementation with infant formula [13,14], positioning and latching, milk supply [14], and poor weight gain [13]. Professional and individualised support strengthens women’s faith in their own ability to breastfeed [15]. When midwives and nurses receive evidence-based training in breastfeeding, it influences their attitudes, knowledge and clinical skills in a positive way [16,17], which increases women’s experience of good breastfeeding support [17] during pregnancy and after birthing [18]. In a study from France, mothers who receive breastfeeding support through preventive visits by health professionals in the first postpartum period more often report fewer breastfeeding problems when the baby is four weeks [19]. In their Cochrane review, Renfrew et al. highlighted the importance of context on treatment effects and that non-proactive support was unlikely to be effective [20]. The systematic review also shows that all forms of extra breastfeeding support influence the duration of breastfeeding positively up to six months after birth [20]. Wambach et al. indicated in their summary of 20 years of evidence, that more research is needed to prevent and treat the most common breastfeeding problem reported by women: insufficient breast milk [11]. This problem is one of complexity and crosses international, cultural, and socioeconomic lines [11]. In a study from Australia mothers who have breastfeeding problems within the first four weeks after birth, more often ended exclusive breastfeeding before the baby was six months old, they also have a shorter total duration of breastfeeding [21]. Mothers in several industrialized countries all too often experience breastfeeding problems such as insufficient breast milk production [8,22-24], and nipples were sore or cracked [8,22-24], in addition health professionals all too often give contradictory advice [24,25]. When health professionals give contradictory counselling, women often feel confused [26,27], and frustrated [27]. When breastfeeding does not proceed as women imagine, health professionals’ emotional support is of importance [28]. In a retrospective case control study from Australia mothers who continue to breastfeed despite problems more often experience good support and counselling from health professionals than those women who end breastfeeding earlier than they wanted [25].

The present study was performed in Sweden, in 2000 - 2003. The overall aim was to investigate whether a process-oriented training intervention within the care team of the antenatal (ANC) and child health centers (CHC) would improve maternal perception of support and strengthen maternal feelings for the baby [29]. These results applied to an understanding of how a process-oriented education in support during childbearing and breastfeeding, for antenatal midwives and postnatal nurses, changed the health care professionals’ attitudes in a positive way. The mothers’ perception of support from the professionals and improved the maternal relationship and feelings for the baby were strengthened compared with the control groups receiving traditional care. There was also a positive correlation between preparation for the parental role and a reduced number of infants being given breast milk substitutes without medical reasons during the first week, as well as a delayed introduction of breast milk substitutes after discharge from hospital, if the health professional received the process-oriented education [18,30-32]. The aim in this study was to evaluate the effects of a process-oriented training in breastfeeding support counselling for midwives and child health nurses, in relation to women’s satisfaction with breastfeeding counselling, problems with insufficient breast milk, pain or nipple sores in relation to exclusive breastfeeding shorter or longer than 3 months.

## Methods

### Design

This is a longitudinal intervention study in which groups of women receive care around childbirth from midwives and child health nurses who have received a process-oriented training program in support during childbirth and breastfeeding, or not. The group of midwives and child health nurses that had not received the process-oriented training program could be considered as the standard care group.

### Setting

The study was performed in a county in the southwest of Sweden. The county consists of 13 municipalities with antenatal and child health centres and comprised of urban, suburban, and rural districts with 280,000 inhabitants. Approximately 2500 births occurred annually at the two hospitals during this time period. The woman and her partner will meet a midwife approximately eight to eleven times during pregnancy. Almost all women give birth in hospital, and care in hospital is provided by midwives who are not previously known to the woman. The average length of hospital stay is between six hours and seven days, and a child-health nurse makes a home visit seven to ten days after the birth, and remains in contact until the baby is old enough to start school at six years of age. At the time of the study, the National Board of Health and Welfare defined breastfeeding as follows: *Exclusive breastfeeding* was defined as breastfeeding with occasional use of water, breast milk substitutes (not more than a few times), and/or solids (not more than one tablespoon per day). *Partial breastfeeding* was defined as infants who received breast milk, and breast milk substitutes (everyday) and/or solids (more than one tablespoon per day). *Total breastfeeding* was defined as the duration of both exclusive and partial breastfeeding [33]. The definition is now revised Sweden in line with WHO definition of breastfeeding [34].

### Intervention

#### ***Phase 1: The process-oriented training program for the midwives and child health nurses in support during childbirth and breastfeeding***

##### 

**Part one** Allocation of municipalities in intervention and control groups. Based on the findings of a baseline study [9,35], the ten largest municipalities in the selected area were paired according to their sizes, and the duration of breastfeeding in those municipalities. For each pair of municipalities, one was then randomly designated to the five-municipality intervention group and one to the five-municipality control group. Furthermore, antenatal midwives and child health nurses were allocated to intervention or control depending on whether their work site had been selected as an intervention municipality or as a control municipality [18,30].

##### 

**Part two** A process-oriented training program [36] in breastfeeding counselling was conducted for the midwives and child health nurses (together referred to as ‘health professionals’ for the remainder of this report) from the intervention municipalities. The process-oriented training program included health professional’s breastfeeding experiences, and breastfeeding attitudes, breastfeeding counselling and communication between antenatal centres and child health centres in line with WHO’s recommendations about breastfeeding support [5] (Additional file 1).

#### ***Phase 2: The sample of women’s and the data collection procedures***

The women included in this study had either been cared for by health professionals in one of the five intervention municipalities or by health professionals in one of the five control municipalities. None of the women knew whether their antenatal midwife and child health nurse had been through the process-oriented training program (intervention groups) or not (control groups). During their stay at the delivery and maternity ward at the hospital, all the women met midwives who had not participated in the process-oriented training program in support during childbirth and breastfeeding.

##### 

**Inclusion criteria** Swedish-speaking, healthy first-time mothers who gave birth to single, healthy full-term babies delivered spontaneously, by vacuum extraction, or by Caesarean section were eligible.

##### 

**Figure 1:**
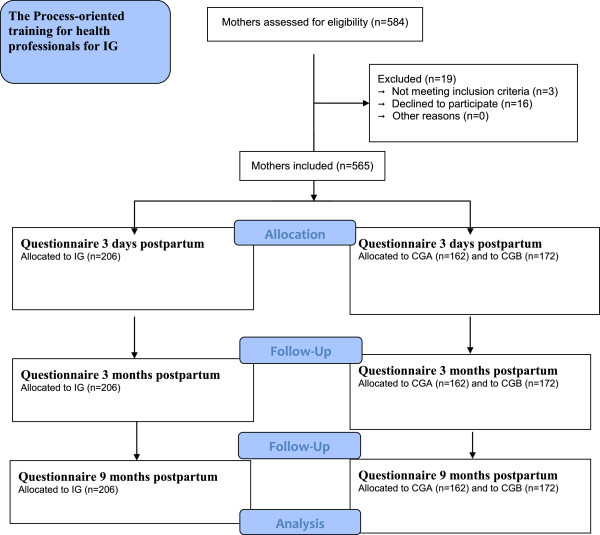
Flow diagram of how mothers enrolled in the Intervention group (IG), Control Group A (CGA) and Control Group B (CGB).

**Exclusion criteria** First-time mothers who had given birth to babies with life-threatening diseases or malformations, for example life-threatening illness such as very severe asphyxia, were excluded.All women who fulfilled the inclusion criteria and had been cared for at the antenatal and child health clinics in the municipalities selected for this study were consecutively identified from the hospital register and asked to participate in the study (n = 584). Of those, 480 gave their informed consent to participate in the study, which translates to a response rate of 82% (Figure 1. Flow diagram).

### Questionnaires

Three questionnaires were developed for this longitudinal study [29] and the questions included in this study are analysed for the first time. Maternity staff members distributed the first questionnaire to the women, who were asked to answer this questionnaire three days after giving birth. Follow-up questionnaires were posted to the women three months and nine months after birthing (Figure 1). Obstetric and demographic data were collected from birth records, and demographic background data were collected when the first questionnaire was administered.

Questions about breastfeeding focused on women’s satisfaction with the breastfeeding counselling, consistent breastfeeding counselling and problems with insufficient breast milk, pain or nipple sores. For example, the questionnaire included questions about planned breastfeeding asked 3 days after birth: “How long do you plan to breastfeed?”, with the answer in months, and questions about breastfeeding problems: “Did you have any breastfeeding problems?” If a woman answered yes, she could indicate more than one problem like insufficient breast milk, pain or nipple sores, mastitis, abscess, fever or other problems, three months after birth. In addition, questions were asked about women’s satisfaction with the breastfeeding counselling and consistent breastfeeding counselling, such as: “Do you feel satisfied with the breastfeeding counselling from the health professionals?” One reminder at each time point was sent to the women who did not respond to the questionnaire.

The three questionnaires developed for this study were pilot-tested by 20 women for acceptability and face validity. In addition, an expert group of midwives and child health nurses was consulted to establish the content validity of the questionnaires. A few minor corrections to the wording were made before the data collection began.

The women who participated in the present study were selected from among those who completed the questionnaire three months after birth, and thus constituted the study of issues of counselling by health professionals. Questions about planned breastfeeding as stated 3 days after birth were collected from the first questionnaire, and data on breastfeeding duration were taken from their answer in the third questionnaire, 9 months after birthing, or by a telephone call, if the breastfeeding rate was longer than 9 months, for women who participated in the study.

### Sample size

The sample size was based on results from the mapping baseline study [9,35] to detect a difference between the IG group and the controls of one month’s in duration of exclusive breastfeeding with β = 0.8 and α 0.05. Before the process-oriented training program commenced, data were collected for a baseline group called Control Group A (CGA, n = 148). Data from CGA were collected before any effects of the intervention could be measured. Data for Control Group B (CGB, n = 160) and Intervention Group (IG, n = 172) were collected simultaneously. Women were divided into two groups depending on whether they exclusive breastfed < 3 months or ≥ 3 months to answer the purpose of the study and issues (Figure 1). This design allowed detection of changes over time and any spill over effects of the intervention. The same five municipalities provided the sample population for CGA and CGB.

### Statistics

For the statistical analyses of the data, we used the Statistical Package for the Social Sciences (SPSS, version 19.0). Central measurements were presented as a mean (M) and dispersion by standard deviation (SD). To test the differences between the groups, one-way ANOVAs and Tukey’s HSD-test for post hoc comparisons were performed. Chi-square tests were performed on category data. Pearson’s rank correlation was used to relate data on the ordinal level. P-values ≤ 0.05 were considered significant [37]. The result is presented with respect to breastfeeding duration less and more than 3 months.

### Ethical considerations

The Ethics Committee of the Medical Faculty of Gothenburg University, Gothenburg, Sweden, approved the study; L 188-99.

## Results

### Response rates, demographic and obstetric data

Response rates for the three questionnaires and the study sample are shown in Table 1. The demographic and obstetric data for the participants and the external dropouts did not differ significantly (data not shown). The response rates for the study were 89% for Questionnaire I (three days after birth), 74% for Questionnaire II (three months after birth), and 69% for Questionnaire III (nine months after birth; n = 540; Table 1). With regard to demographic and obstetrical data, no significant differences existed between the women in the IG compared to the women in the CG (Table 2).

**Table 1:** Response rate for all groups at 3 days, 3 months, and 9 months postpartum

	**IG N = 206**		**CGA N = 162**		**CGB N = 172**		**Total N = 540**	**p**
**3 days postpartum, n (%)**	172	(84%)	148	(91%)	160	(93%)	480 (89%)	n.s
**3 months postpartum, n (%)**	145	(70%)	126	(78%)	132	(77%)	403 (74%)	n.s
**9 months postpartum, n (%)**	131	(64%)	116	(72%)	125	(73%)	372 (69%)	n.s

The intervention group (IG), Control Group A (CGA) and Control Group B (CGB).

**Table 2:** Sociodemographic and obstetric data for mothers in all groups at three days after birth

	**IG 172**		**CGA 148**		**CGB 160**	
**Age in years** (m and SD)	26.6	(4.5)	27.2	(4.6)	27.0	(5.0)
**Gestational weeks** (m and SD)	40.4	(1.4)	40.5	(1.4)	40.4	(1.4)
**Education**						
Compulsory school (%)	6	(3%)	5	(3%)	3	(2%)
High school (%)	77	(37%)	73	(45%)	71	(41%)
University (%)	74	(36%)	55	(34%)	62	(36%)
Other (%)	14	(7%)	15	(9%)	21	(12%)
Missing	35	(17%)	14	(9%)	15	(9%)
**Marital status**						
Cohabitation (3 days postpartum)	125	(61%)	102	(63%)	118	(69%)
Married	42	(20%)	43	(27%)	38	(22%)
Single	3	(1.5%)	2	(1%)	2	(1%)
Other	1	(0.5%)	3	(2%)	2	(1%)
Missing	35	(17%)	12	(7%)	12	(7%)
**Obstetric data**						
Vaginal delivery (%)	146	(70%)	120	(74%)	129	(75%)
Caesarean section (%)	32	(16%)	22	(14%)	31	(18%)
Vacuum extraction/forceps (%)	28	(14%)	20	(12%)	12	(7%)

The intervention group (IG), Control Group A (CGA) and Control Group B (CGB).

### Women’s’ planned exclusive and total breastfeeding three days after birth compared with the outcome of exclusive breastfeeding duration < 3 months and ≥ 3 months

Women who had *exclusive breastfeeding duration* ***≥*** *3 months* more often breastfed as long as they had planned, compared with women who had *exclusive breastfeeding duration < 3 months,* who more rarely breastfed as long as they had planned. There were no significant differences between IG and the control groups (Table 3).

**Table 3:** Mothers’ planned breastfeeding duration, breastfeeding satisfaction, counseling, problems and duration, in all groups

	**IG = 145**			**CGA = 126**			**CGB = 132**			**IG/CGA**	**IG/CGB**	**CGA/CGB**
**Exclusive breastfeeding <3 months**	**N = 30**	**m**	**SD**	**N = 31**	**m**	**SD**	**N = 35**	**m**	**SD**	**p value Tukey’s HSD test**		
Planned exclusive breastfeeding	7	5,5	0,9	12	5,3	1,1	13	5,3	1,1	0,942	0,949	1,000
Planned total breastfeeding	5	7,2	2,5	6	9,8	3,0	4	7,0	0,8	0,220	0,992	0,214
**Exclusive breastfeeding <3 months**	**N = 30**	**m**	**SD**	**N = 31**	**m**	**SD**	**N = 35**	**m**	**SD**	**p value Tukey’s HSD test**		
Exclusive breastfeeding	30	1,5	0,8	31	1,1	0,8	35	1,2	0,9	0,208	0,553	0,752
Total breastfeeding	27	2,6	1,8	30	3,2	3,0	30	3,2	2,5	0,639	0,646	1,000
**Exclusive breastfeeding <3 months**	**N = 30**	**n**	**(%)**	**N = 31**	**n**	**(%)**	**N = 35**	**n**	**(%)**	**p value Pearson Chi-square test**		
Satisfaction with the breastfeeding counseling	20	15	75	27	8	30	30	16	53	0,008**		
Coherent breastfeeding counseling	20	18	90	24	10	42	28	21	75	0,002**		
Perception of insufficient breast milk	20	4	20	27	15	56	30	18	60	0,01*		
Pain in the breast/nipple	20	6	30	27	6	22	30	4	13	0,354		
Nipple sores	20	8	40	27	13	48	30	11	37	0,671		
**Exclusive breastfeeding = > 3 months**	**N = 105**	**m**	**SD**	**N = 71**	**m**	**SD**	**N = 75**	**m**	**SD**	**p value Tukey’s HSD test**		
Planned exclusive breastfeeding	40	5,6	1,6	30	6,9	6,5	31	5,7	0,6	0,300	0,994	0,395
Planned total breastfeeding	25	8,8	2,6	26	8,8	2,5	32	8,2	2,1	0,997	0,580	0,624
**Exclusive breastfeeding = > 3 months**	**N = 105**	**m**	**SD**	**N = 71**	**m**	**SD**	**N = 75**	**m**	**SD**	**p value Tukey’s HSD test**		
Exclusive breastfeeding	105	5,0	1,0	71	4,9	1,1	75	5,0	0,9	0,742	1,000	0,773
Total breastfeeding	98	7,4	2,6	64	7,8	3,6	62	7,4	3,2	0,636	0,985	0,783
**Exclusive Breastfeeding = > 3 months**	**N = 105**	**n**	**(%)**	**N = 71**	**n**	**(%)**	**N = 75**	**n**	**(%)**	**p value Pearson Chi-square test**		
Satisfaction with the breastfeeding counseling	73	57	78	57	47	82	66	52	79	0,812		
Coherent breastfeeding counseling	67	53	79	56	43	77	63	45	71	0,581		
Perception of insufficient breast milk	73	15	21	57	9	16	66	12	18	0,784		
Pain in the breast/nipple	73	17	23	57	5	9	66	9	14	0,067		
Nipple sores	73	25	34	57	13	23	66	28	42	0,071		

The intervention group (IG), Control Group A (CGA) and Control Group B (CGB). P-value: ≤0.05 = * and <0.01 **.

### Women’s satisfaction with the breastfeeding counselling, with an exclusive breastfeeding duration < 3 months and ≥ 3 months

Women in the IG group, with an *exclusive breastfeeding duration < 3 months,* were more satisfied with the breastfeeding counselling from the health care professionals compared with the women in the control groups (p = 0.008; Table 3). Women’s satisfaction with the breastfeeding counselling from the health care professionals showed no significant differences between IG and the control groups for women with *exclusive breastfeeding duration* ***≥*** *3 months* (Table 3).

### Women’s satisfaction with a coherent breastfeeding counselling, with an exclusive breastfeeding duration < 3 months and ≥ 3 months

Women in the IG group, with an *exclusive breastfeeding duration < 3 months,* were significantly more satisfied with coherent breastfeeding counselling compared with the women in the control groups (p = 0.002; Table 3). The results showed no significant difference between IG and control groups for women with *exclusive breastfeeding duration* ***≥*** *3 months* (Table 3).

### Women’s breastfeeding problems, with an exclusive breastfeeding duration < 3 months and ≥ 3 months

There were fewer women with *exclusive breastfeeding duration < 3 months* who experienced insufficient breast milk production that ended their breastfeeding during the first three months in the IG compared with the control groups (p = 0.01; Table 3). No significant difference was observed between the IG and control groups regarding pain in the breast/nipple or nipple sores for women with *exclusive breastfeeding duration < 3 months* (Table 3). The results showed no significant difference between IG and control groups regarding the number of women who experienced insufficient breast milk production, pain in the breast/nipple or nipple sores for women with *exclusive breastfeeding duration* ***≥*** *3 months* (Table 3).

## Discussion

The main findings of this study showed that women who received support and counselling from health professionals who had received a process-oriented training in support during breastfeeding increased their ability to succeed with breastfeeding. Women in the intervention group (IG), with exclusive breastfeeding duration < 3 months, were more satisfied with coherent counselling from the health professionals, despite not breastfeeding as long as they planned, compared with women in the control groups. In addition, there were fewer women in the IG with breastfeeding problems such as experienced insufficient breast milk production, compared with women in the control groups.

Many of the women in this study planned their exclusive breastfeeding in line with WHO recommendations about exclusively breastfeeding for six months [5]. In contrast the result of this study showed that women did not always breastfeed as long as they have planned. If the exclusive breastfeeding was shorter than three months they often ended the breastfeeding earlier than they planned, perhaps even before breastfeeding was established. Results from another studies also shows that two-thirds of mothers who intend to exclusively breastfeed are not meeting their intended duration [38].

Our result are in line with other research showing that when health professionals receive breastfeeding education based on WHO guidelines, they feel more secure and experience an increased ability to support women with coherent, evidence-based counselling [39]. Another study shows that when caregivers have communication skills, their ability to empathize and find individual solutions increases, which reduces the risk that women perceive the advice as contradictory [26]. Hence, the health professionals in the IG offered women individualized support, and it resulted in increased confidence in breastfeeding, compared with the women in the control group. Women need to receive realistic, consistent and evidence-based information on breastfeeding during pregnancy [27]. It has been found that women with higher knowledge of breastfeeding have more confidence in their ability to breastfeed [40]. A previously published study from this data set showed that this kind of education for health professionals in support during childbirth and breastfeeding increased women’s experience of professional support during pregnancy and after birth [18]. In addition, the results may be due to the women having better knowledge and more realistic expectations about breastfeeding, which may have increased their confidence in solving breastfeeding problems. Studies show when women have doubts about their own ability to breastfeed, contradictory advice has a more negative impact for them [13,14]. These results may also affect women’s ability to manage their breastfeeding problems better by themselves, depending on whether the breastfeeding counselling was more suited to the women’s needs and their life situation.

Further, it was found that there were significantly fewer women who experienced insufficient breast milk production in the IG compared with the control groups, for women who had an exclusive breastfeeding duration < 3 months. The reason why these women ended breastfeeding before they planned three days after birth may due to other reasons than those considered in the present study. When professionals are trained in line with the WHO guidelines, the breastfeeding support to women increases, and the women also feel more comfortable in their experience of having enough breast milk production [41]. The results from this study also showed that women who breastfed exclusively **≥** 3 months and experienced insufficient breast milk production were satisfied with their professional counselling, in both the IG and control groups. Women are often unsure about their ability to breastfeed [15], and up to 50% report the perception of insufficient breast milk production [8]. Despite women’s experience of insufficient breast milk production, only about five percent have a biological factor making them unable to produce enough breast milk [7,8]. Most women who experience insufficient breast milk production provide infant formula, but some women choose to latch the baby on to stimulate the breast or to seek advice from health professionals [42,43]. Dykes and Williams reported that women with experience of insufficient breast milk were dissatisfied with incorrect and conflicting advice from health professionals, and it had negative consequences for their ability to breastfeed [44]. These results may due to the fact that women in IG who experienced insufficient breast milk production received counselling from health professionals to breastfeed on demand, resulting in stronger self-esteem, or vice versa. When professional breastfeeding support began during pregnancy and continued after birth and when breastfeeding was established, it increased women’s confidence in their ability to breastfeed and solved breastfeeding problems, which led to longer breastfeeding duration. Women with support from health care professionals with the process-oriented training were satisfied with their professional counselling and motivated and able to solve their breastfeeding problems.

This longitudinal intervention method with two control groups (CGA data was collected before any effects of the intervention could be measured) was selected as being suitable for the study. This is a design suggested to measure possible spill over effects [45]. More differences were found when the IG was compared with the CGA than when the IG was compared with the CGB (where data were collected simultaneously with the IG). The results show that changes also take place among controls when an intervention is being rolled out. In the professional network of midwives and child health nurses, knowledge and information are shared, which easily leads to spill over effects between intervention and control professionals. These results thus demonstrate the value of using a historic control group, which will provide a baseline against which to evaluate the spill over effect.

Midwives at antenatal centres need a better understanding about their important role in breastfeeding counselling during women’s pregnancy. This could help women become better prepared for breastfeeding and give them more realistic expectations of breastfeeding. Since many women are worried about not being able to produce enough breast milk, it is important to increase women’s confidence in their ability to breastfeed. Health professionals should emphasize proximity and the relationship between women and their baby and avoid asking questions about sufficient breast milk production. Encouragement is a powerful way to support breastfeeding, and it increases women’s confidence in their ability to breastfeed.

## Conclusions

A process-oriented training for health professionals’ support influenced women’s ability to solve breastfeeding problems such as the perception of insufficient breast milk production in a positive way. Women with exclusive breastfeeding duration ≥ 3 months more often had a breastfeeding duration in conformity with their planned breastfeeding duration, compared with women who had a breastfeeding duration < 3 months.

## Competing interests

The authors declare that they have no competing interests.

## Authors’ contributions

AE participated in the study design and collected the data. AE, IB and LBM analyzed the data and drafted the manuscript. All authors read and approved the final manuscript.

## Supplementary Material

Additional file 1 The process-oriented training program for health professionals. Click here for file
