# Supplementary material for: Process-oriented training in breastfeeding for health professionals decreases women’s experiences of breastfeeding challenges
Source: Int Breastfeed J. 2014 Sep 9;9:15. doi: 10.1186/1746-4358-9-15 (PMC4163059; doi:10.1186/1746-4358-9-15)
Supplement: Additional file 1 — The process-oriented training program for health professionals. [file 1746-4358-9-15-S1.doc]

**The process-oriented training program for healthcare professionals**

**Definition of the process-oriented training program:** In order to change the attitudes of health professionals the training program was based on literature reviews and collegial discussions containing professional stance, reflective processes, problem-solving processes, and practical skills in relation to support during childbearing. To ensure that the healthcare professionals would be ready to meet the demands of their profession they were trained in problem solving, self-reflection, and decision-making (in terms of competence), and their personal qualifications evaluated (Jerlock, Falk & Severinsson (2003)).

The process-oriented training program involved seven days of lectures and its main theme focused on the participants’ breastfeeding experiences (both private and professional), breastfeeding attitudes, breastfeeding counselling, and collaboration and communication between antenatal centers and child health centers in line with WHO recommendations.* Midwives and postnatal nurses were asked to reflect on different areas relating to breastfeeding support. The training program supervisors were chosen to strengthen the process between the healthcare centers and the hospital wards. Both the literature and the lecturers raised parenthood, breast-feeding, relations, attachment, the baby’s ability as well as complications around childbirth as well as a common parental education with both midwives and postnatal nurses included. It was the same parental education groups during pregnancy and the babies first year of life

The healthcare professionals chose the following topics as homework:

- - How do we protect, promote, and support breastfeeding?
  - How do we inform mothers about parenthood and family life?
  - How do we broaden our minds in order to help parents from other cultural backgrounds?
  - How can parental leave be shared on an equal basis or should we not do this?
  - What is attachment and how do parents’ best support attachment?
  - What happens if postnatal depression occurs?
  - How do we strengthen the relationships between healthcare professionals and their significant others?
  - How do we support the families that experience complicated deliveries?
  - How do we best support parent-infant interaction when the infant is cared for on the neonatal ward?
  - How do we talk about lifestyle problems?
  - How do we approach single parents?

Jerlock M, Falk K, Severinsson E. 2003 Academic nursing education guidelines: Tools for bridging the gap between theory, research and practice. Nurs Health Sci 5: 219-228.

Additional file 1: The process-oriented training program for healthcare professionals

*WHO, UNICEF. Protect, Promoting and Supporting Breastfeeding. Geneva: World Health Organization; 1989.
